# Healthcare providers’ experiences in hospital resuscitation of patients with COVID-19: a qualitative study

**DOI:** 10.1186/s12912-022-01020-y

**Published:** 2022-08-25

**Authors:** Afshin Goodarzi, Masoud Khodaveisi, Alireza Abdi, Rasoul Salimi, Khodayar Oshvandi

**Affiliations:** 1 Department of medical emergency, Faculty of Paramedics, Kermanshah University of Medical Sciences, Kermanshah, Iran; 2 Chronic Diseases (Home Care) Research Center, Department of Community Health Nursing, Hamadan University of Medical Sciences, Hamadan, Iran; 3 Department of Nursing, School of Nursing & Midwifery, Kermanshah University of Medical Sciences, Kermanshah, Iran; 4 Department of Emergency Medicine, School of Medicine, Besat Hospital, Hamadan University of Medical Sciences, Hamadan, Iran; 5 Mother and Child Care Research Center, Nursing and Midwifery School, Hamadan University of Medical Sciences, Hamadan, Iran

**Keywords:** COVID-19, Cardiopulmonary resuscitation, Qualitative research, Experiences

## Abstract

**Background:**

The COVID-19 epidemic has globally challenged medical practices, including cardiopulmonary resuscitation (CPR). Numerous challenges affect healthcare providers (HCPs) who are members of the resuscitation team and the resuscitation process in COVID-19 patients. As a result, HCPs may experience different dilemmas about CPR. Failure to recognize these experiences can harm both HCPs and patients. This study aimed to explore the HCP’s experiences of CPR in patients with COVID-19.

**Methods:**

A qualitative study was conducted using semi-structured interviews with 26 participants in the emergency departments of Besat, Golestan, and Imam Reza hospitals (in the west of Iran) using the hermeneutic phenomenology approach. The data were analyzed using the 6-step Smith interpretative phenomenological analysis (IPA) method.

**Results:**

The mean age of the participants was 38 years. Most of them (61.5%) were male and had a Bachelor’s degree in nursing (46.1%).The data analysis resulted in extracting four super-ordinate and nine sub-ordinate themes. “Human aspects of Care”, “Perceived Psychological Effects of Resuscitation in COVID-19”, “HCP’s perceptions of factors affecting the resuscitation process in COVID-19”, and “Perceived differences in COVID-19 resuscitation compared to non-COVID patients” were super-ordinate themes.

**Conclusions:**

The participants experienced a wide range of difficult feelings and emotions while resuscitating the patients with COVID-19, suggesting the effect of the COVID-19 epidemic on HCPs and the resuscitation process. They experienced stress and fear, and the resuscitation process was influenced by their compassion, underlying patient conditions, resuscitation futility, and participants’ fatigue or lack of oxygen due to the use of personal protective equipment (PPE).

## Background

The World Health Organization (WHO) declared the Severe Acute Respiratory Syndrome Pandemic (SARS-CoV-2) on March 11, 2020. On December 10 of 2021, about 2 years after reporting the first case of COVID-19 and after a global estimate of 268 million infections and 5.3 million deaths, omicron strain was reported as a new SARS-CoV-2 variant of concern (VoC) [1, 2]. The omicron emerged when tired healthcare systems were under intense pressure and grueling stress from previous waves of the COVID-19 pandemic. Therefore, scientists are highly concerned about the high transmission power of omicron and its greater resistance to available vaccines [3].

The high mortality of COVID-19, the disease transmission capacity, and the shortcomings of health facilities [4], have had a significant impact on the employees’ mental health [5] and globally confronted the medical systems to some challenges [6]. In this respect, cardiopulmonary resuscitation (CPR) is one of the key components related to emergencies challenged by the high prevalence of COVID-19 mortality [7]. While there are some controversies about increasing aerosol production due to chest compression during resuscitation, there is strong evidence for increased aerosol production through procedures including positive pressure ventilation, suctioning, and the establishment of an advanced airway [8–11]. During these stages, viral particles can remain suspended in the air for half-life an hour and be inhaled by people close to the patient. Additionally, resuscitation efforts require multiple providers working close to one another and the patient [8, 12]. Such characteristics make resuscitation a risky procedure, affecting the healthcare providers (HCPs) and resuscitation outcomes.

Reports show that the COVID pandemic has affected CPR and its outcomes, leading to poor resuscitation outcomes in patients with COVID-19. For instance, a study in China [13], reported that the primary success rate of CPR was 13.2% and 30-day survival rate was 2.9%. According to the findings of two studies on in-hospital and out-of-hospital cardiac arrest, none of the patients was discharged alive after successful initial resuscitation [7, 14]. Elsewhere, the present study’s authors [15] showed that the primary success rate of CPR was 9% and survival to discharge rate was 2%.

Although poor results in the resuscitation of patients with COVID-19 have been linked in some studies to the inherent fatality of the disease [7, 14], the high-risk nature of resuscitation for HCPs highlights the need for further investigation into the human factors involved in resuscitation. The results of COVID-19 studies show that the provision of care, especially in the front lines, has led to different physical and mental experiences for health care providers. In one study, high stress levels, depression, losing control over issues, fear, and anxiety were associated with care delivery [16]. However, in other studies, fatigue, helplessness, resilience challenges and altruistic acts [17, 18] have been extracted as the main themes affecting the HCPs and their care delivery. However, no study has been done on resuscitation team members in this regard.

Close and prolonged contact with the critical condition of cardiac arrest in patients infected by COVID − 19 has different emotional effects hidden behind the masks of HCPs. Failure to recognize them can negatively affect resuscitation team members and resuscitation outcomes. The only way to understand what resuscitation team members have gone through is to reconstruct the live experiences of them. Thus, according to the research question, i.e., “meaning of resuscitation team members’ experiences”, this study aimed to explore the HCPs’ experiences of cardiopulmonary resuscitation in patients with COVID-19.

## Methods

### Research design

This study was a part of a doctoral dissertation conducted 1 year after the outbreak of COVID-19. In this research, a qualitative study was conducted using semi-structured interviews with participants, during the COVID-19 crisis. The interviews were conducted face-to-face with the presence of researchers and participants and without the presence of a third party, from February to August 2021. The main research question was to seek the “meaning of resuscitation team members’ experiences of resuscitation of patients with COVID-19”. The activity of the researchers of the present study in the field of resuscitation makes it impossible to complete bracketing, and creates the ground for the application of the interpretative phenomenological analysis (IPA) for data analysis. Bracketing is a method used in qualitative research to mitigate the potentially deleterious effects of preconceptions that may taint the research process. In an interpretive phenomenological study, complete bracketing does not occur [19]. The IPA focuses on identifying, analyzing, and interpreting patterns of meaning within qualitative data. Such a method allows for a rich in-depth exploration of participants’ experiences [20, 21]. In this way, participants create their emotions from experiences (the first hermeneutic layer), and the researcher makes his interpretation based on them (the second hermeneutic layer). IPA is essentially committed to examine closely the unique, particular experience of each individual participant, from which themes that respond to the research question(s) emerge [21].

### Participants

The participants included resuscitation team members in the emergency departments of Besat, Golestan, and Imam Reza teaching hospitals in the west of Iran. From the epidemic’s beginning, the mentioned centers received patients with COVID-19. The inclusion criteria were being a physician, nurse, nurse anesthetist, and nurse in the role of supervisor, who worked directly with confirmed COVID-19 cases and had at least 5 cases of attending the resuscitation of cardiac arrest patients with COVID-19. Phenomenology uses criterion sampling, in which participants meet predefined criteria. The most prominent criterion is the participant’s experience with the phenomenon under study. The researchers look for participants who have shared an experience, but vary in characteristics and in their experience modalities [22]. Thus participants were selected through purposive sampling, and a maximum variation sampling strategy was chosen to capture all groups’experiences. The main variation variables included gender, age, job title, work experience, and responsibility in the resuscitation team. Data gathering and analysis were conducted concurrently until data saturation (i.e.,when maximum information was collected about the phenomenon under study and generally depends on the richness of the experiences) [22, 23]. It should be noted that all the people invited to the study participated in the interview and none of the interviews needed to be repeated. Data saturation was reached after interviewing 24 participants; however, two more interviews were conducted to ensure data sufficiency. Overall, 26 health care providers who were members of the resuscitation team participated in the study.

### Data collection

The main method of data gathering was semi-structured in-depth interviews. The researchers designed the initial format of semi-structured interview at the beginning of the study; however, it was modified for each interview based on the analysis of the previous interviews. Interviews were conducted in workplace, outside the working hours and with the participants’ permission. Due to the work experiences of the researchers of the current study in the field of resuscitation, there was a professional relationship between the researchers and some of the participants prior to study. Moreover, before each interview, we explained the topic and aims of the research. Each interview lasted 45 to 67 minutes. The interviews were conducted in Persian and then translated into English. Interviewers (AG,MK, and ARA) were faculty members (Ph.D.) who were male and experts in qualitative studies. Demographic information of study participants was obtained before the interview. The interview began with an open-ended question, “talk about your experiences with resuscitation in COVID-19” and “express your understanding of resuscitation in COVID-19 patients”. Following the interview, the researcher asked questions based on the participants’ speeches and the purpose of the study: “How does resuscitation in COVID-19 patients affect you?”, “can you talk about your feelings in this situation?” and “What did you feel when you are in the resuscitation of a patient with COVID-19? At the end of the interview, the participants were asked if they had anything to add. Interviews were recorded on an MP3 player and verbatim transcribed by the first author as soon as possible.

### Data analysis

The 6-step IPA method introduced by Smith was used for data analysis [21, 24]. The steps of this method include: 1) reading and re-reading, 2) initial note-taking, 3) developing emergent themes, 4) searching for connections across emergent themes, 5) moving to the next case, and 6) looking for patterns across cases. The first and second steps were performed by the researchers involved in the first interview data. For this purpose first interview was listened to several times and then turned into a transcribed text. Transcribed interviews were sent to interviewees to check for their accuracy. The transcripts were read from start to end several times by the corresponding author and interviewers. Reading and re-reading the interviews caused more engagement of the researchers with the data. Free notes were taken when studying the texts. These notes reflected the researcher’s initial thoughts and observations to encode in the text. In this way, the researchers extracted important statements, and each statement was labeled with a code. In the third stage, similar codes and ones making a pattern were summarized in to sub-ordinate themes. The super-ordinate themes emerged in the fourth stage by determining the hierarchical relationship between the sub-ordinate themes. The fifth stage was moving on to the next case. In this way, after the next interview, the first four analysis steps were repeated, and by extracting new data, previously super-ordinate themes were developed, or new themes were extracted. In the sixth step, super-ordinate common themes were developed for all 26 participants in the study, followed by identifying the main patterns across all cases. This step was not the last data analysis step, and the analysis was continued even while recording the results. MAXQDA10.0R250412 software was used to manage the data.

### Data rigor

The researchers ensured the data credibility through deep and long involvement with the data and member checking. The transcripts were read from start to end several times by the corresponding author and interviewers to immerse in the texts and fully understand them. The transcripts were returned to participants for comment and/or correction. Also, after analyzing the data, the study results were presented to 10 participants, who agreed with the main patterns observed. Several interviews were chosen randomly to check the data’s confirmability, the purposes of the study, codes, and themes were also given to two experts in the field of qualitative studies who were not part of the study team, and their advice was used. Three study team members with sufficient experience in qualitative study analysis performed the coding steps separately to determine the data’s dependability. The necessary agreement was reached in this process by discussing data analysis in several sessions. The present study confirmed the data transferability using various sampling from different people in the resuscitation team. Also, the characteristics of the research population and the research process were described clearly and accurately to make it possible to follow the research path. Furthermore, publishing the findings and readers’ judgment will help determine the data transfer capability.

## Results

Among the 26 participants in the present study, 11 cases (42.3%), 8 cases (30.8%), and 7 cases(26.9%) were in the emergency resuscitation teams of Imam Reza, Besat, and Golestan hospitals, respectively. The mean age of the study participants was 38 years (24–53 years), and most of them (16 cases, 61.5%) were identified as male. The mean work experience of the study participants was 12.8 years (2–27 years), and most of them had a Bachelor’s degree in nursing (12 cases, 46.1%). Table 1 summarizes the other characteristics of the participants.

**Table 1:** Characteristics of the participants

Variable	Frequency	Percent
Job title		
Nurse	16	61.5
Physician	6	23.1
Nurse anesthetist	**4**	15.4
Posittion in the resuscitation team		
Leader	6	23.1
supervisor	6	23.1
Airway management	4	15.4
Chest compression, Drug injection or shock discharge	10	38.4
Educational level		
BSc of nursing	12	46.1
MSc of nursing	4	15.4
BSc of nurse anesthetist	4	15.4
Emergency medicine specialist	5	19.2
GP	1	3.9
Gender		
Female	10	38.5
Male	16	61.5
Marital status		
Single	10	38.5
Married	16	61.5

Data analysis led to the extraction of 161 codes related to resuscitation experiences in patients with COVID-19. Overall, 4 super-ordinate and 9 sub-ordinate themes were extracted from these experiences:

1. The human aspect of care, with two sub-ordinate themes: “care with compassion in resuscitation” and “failure to observe self-protection for speed of action in resuscitation”.

2-. Perceived psychological effects of resuscitation in COVID-19, with two sub-ordinate themes: “Stress transmitting the disease to family members” and “Fear of contamination during resuscitation”.
3-. HCPs’ perceptions of factors affecting the resuscitation process in COVID-19, with two sub-ordinate themes: “Underlying conditions with COVID-19” and “effect of pulmonary involvement on resuscitation”.
4-. Perceived differences in COVID-19 resuscitation compared to non-COVID patients, with three sub-ordinate themes: “Personal protective equipment (PPE) is a limit to effective resuscitation in COVID-19“, “Delay in the resuscitation”, and “The futility of resuscitation in COVID-19”.

### The human aspect of care

The first super-ordinate theme in this study was the human aspects of care, which was extracted from statements of six participants, and 23 codes were obtained in this regard. Participants stated that resuscitation of patients with COVID-19 is done with more compassion, leading to more resuscitation efforts. Because of concerns for the patient’s survival, participants sometimes prefer rapid resuscitation to their personal protection. Two sub-ordinate themes were obtained in this regard:

#### Care with compassion in resuscitation

The first sub-ordinate theme of the human aspects of care was “Care with compassion in resuscitation”, which was mentioned by four participants. Based on this theme “participants” recall their own experiences of being infected with COVID-19, make emotional connections with COVID-19 positive patients, and subsequently, they revealed compassion when they need CPR. In this respect, a 49-year-old nurse with 22 years of experience in various departments, including the resuscitation team, said about understanding the condition of COVID-19 patients in the final stages:

*‘You know that you are dying, your breath does not come up, and you cannot do anything. I had a period of shortness of breath when I got Corona, and I understand them. I try to do everything I can; It really makes me cry’. (No. 15 participant).*

Also, one of the supervisors, a 39-year-old woman with 16 years of experience in the nursing profession, said in a tone full of compassion:

*‘It’s really sad to see someone who was completely healthy until yesterday and now needs resuscitation, I try to do everything I can for her or him, maybe she or he will come back to life’. (Participant No. 13).*

Compassionate care allowed staff to do everything to help the patient, even prolonging the resuscitation effort to achieve better results.

#### Failure to observe self-protection for speed of action in resuscitation

“Failure to observe self-protection for speed of action in resuscitation”, mentioned by four participants, was the second sub-ordinate theme of the human aspects of care. When the HCPs encountered a patient with cardiac arrest, their sense of humanity made them prefer to help the critically ill patient rather than protect themselves. A 25-year-old nurse anesthetist with three years of experience in the emergency resuscitation room said:

*‘Well, we have to observe personal protection; but in many places, I did not do it in an emergency and resuscitated the patient with a mask or shield. I feel better this way. It isn’t possible both think about the save the patient’s life and spend time for using PPE; there isn’t time for patients who need to be resuscitated upon arrival in the emergency room’. (Participant No. 17).*

One of the 49-year-old nurses, who seemed to give up their safety in order not to waste time, said with pride of this process:

*‘I have been accustomed for years to think about helping the patient immediately in resuscitation; I can’t spend much time being ready for resuscitation. It is really hard to see a patient is suffering, and you are thinking of protecting yourself more’. (participant No. 15).*

Participants felt better about not losing time in resuscitation to protect themselves.

### Perceived psychological effects of resuscitation in COVID-19

The second super-ordinate theme in this study was the perceived psychological effects of resuscitation resulting from the statements of 10 participants. Overall, 50 codes were obtained for this theme. Because of their close and prolonged contact with the Patient under resuscitation, the participants expressed concern about becoming infected with COVID-19 or transmitting the disease to family members. Two sub-ordinate themes were discovered in this regard.

#### Stress transmitting the disease to family members

Seven participants mentioned “stress transmission the disease to family members” as the first sub-ordinate theme of perceived psychological effects. The participants experienced stress after attending the resuscitation of patients because of their history of transmitting the virus to family members and their concerns and consequences. In this regard, a 26-year-old nurse anesthetist with 4 years of experience, two of which were related to the emergency resuscitation team, said:

*‘There is anxiety and stress in resuscitating a patient with corona disease, even in patients who are suspected of corona disease. During resuscitation, contact with this patient is very close. After resuscitation, I always change all my work clothes in the parking when I go home. I take a shower immediately; I even restrict my contact with family members for a few days to see if I have any symptoms’. (Participant No. 20).*

A 28-year-old nurse with 4 years of experience in the emergency department said:

*‘COVID-19 has infected me twice previously*. *I didn’t know the first time until my body began to ache, and I had infected my wife during this time. She also infected her sister and children. I was always worried that something terrible would happen to someone. Well, the contact is very close in resuscitation, and I am worried about transmitting the virus home’. (Participant No. 26).*

The stress of infecting family members made the participants more sensitive to the removal of possible contamination after attending to the resuscitation of these patients, and they restricted contact with family members for a while.

#### Fear of contamination during resuscitation

“Fear of contamination during resuscitation” was the second sub-ordinate theme of perceived psychological effects, addressed by nine participants. The frequent presence in the resuscitation of COVID patients, combined with some participants’ experience of uncomplicated infection, made them less fearful of attending COVID-19 resuscitation than the onset of the epidemic. However, some participants, despite being vaccinated, still felt the nature of resuscitation was dangerous. In an interview with a 39-year-old supervisor, she explained with a calmness that may have resulted from the increased experience of dealing with COVID-19 patients:

*‘Because the disease was unknown for the first few months, there was some fear of transmission during resuscitation. Suddenly, there was a rumor that we thought whoever got the disease would die right away’. (Participant No. 13).*

Another nurse, this one with 16 years of experience, added:

*‘Despite the vaccination, I am still worried about being infected during resuscitation with these new strains. The most likely infection for nurses is during resuscitation. The rest of my colleagues are also worried. Our contact in resuscitation is much closer. We work directly with the patient’s respiratory secretions’. (Participant No. 21).*

Participants who were afraid of the patient’s resuscitation did not want to prolong resuscitation in these patients. They even thought that measures like repeated suctioning, which increased the risk of contamination spreading during resuscitation, were unnecessary. In this respect, one of the nurses on the resuscitation team with 4 years of experience in the emergency department said:

*‘Even the staff maintains a distance from the patient during chest compression****.*** *Due to the fear of making contact with the patient, even the principles of proper chest compression are not followed. They wait for the doctor at any moment to tell them not to continue’. (Participant No. 26).*

### HCPs’ perceptions of factors affecting the resuscitation process in COVID-19

The third super-ordinate theme in this study was perceived factors affecting resuscitation, which was based on statements from 9 participants and resulted in 32 codes. Based on this theme, the participants stated that the resuscitation process is affected by the patients’underlying conditions and the level of pulmonary involvement. Based on this theme, two sub-ordinate themes were observed in this regard.

#### Underlying conditions with COVID-19

“Underlying conditions with COVID-19”, noted by 5 participants, was the first sub-ordinate theme of the factors affecting the resuscitation process in COVID-19. The experiences of participants showed that the quality and quantity of resuscitation in patients with Covid-19 are affected by patients’ old age and their underlying disease. One of the supervisors, with 39 years of age and 16 years of work experience in various departments (including the emergency department), while he complained about the involvement of underlying conditions in the resuscitation process, said:

*‘As an observer, I checked the patients’ code and found out that the chest compression was stopped in the newly coded patient. What are you doing? Why are you not resuscitating? Why are you not doing chest compression? I said. Dr. S. told us to stop chest compression, they said. The code has been paged for 1 min, I said. The 70-year-old patient has an underlying disease; he will not be alive anymore, they said’. (participant number 18).*

Also, an emergency medicine specialist with 33 years of age and 4 years of experience in the emergency department, as the team leader said:

*‘A patient had diabetes for several years and had kidney failure, according to the medical history. This issue has an impact on one’s mentality. I’m not saying we don’t resuscitate, but the quality of resuscitation differs significantly from that performed in the absence of an underlying problem’. (No. 7 participant).*

The presence of underlying conditions in resuscitation affected the quantity and quality of resuscitation in these patients such that only a few resuscitation cycles were performed in some cases.

#### Effect of pulmonary involvement on resuscitation

“Effect of pulmonary involvement on resuscitation”, mentioned by six participants, was the second sub-ordinate theme of the participants’ perceptions of the factors affecting the resuscitation process in COVID-19. According to this theme, if the HCPs were aware of the high severity of the patient’s lung involvement, they would be subconsciously discouraged from resuscitation. A 42-year-old nurse who has been working in the resuscitation team of these patients in the emergency department since the beginning of the COVID-19 epidemic said:

*‘Usually, patients with oxygen saturation less than 50% rarely respond to resuscitation. However, we had a patient whose oxygen saturation reached 30% due to lung involvement, but he survived. Nevertheless, I feel that prior information of a lung CT scan affects the resuscitation process even for myself subconsciously’. (Participant No. 14).*

Another 36-year-old nurse, who had 6 years of experience in the resuscitation team, mentioned:

*‘My colleagues’ mentality from CT scans of the patient’s lungs affects the resuscitation process. When a patient gets a CT scan, they say, Oh, how bad the lung involvement is. The same patient does not have much quality resuscitation when he needs resuscitation’. (Participant No. 11).*

According to participants’ experiences, the mentality derived from the level of pulmonary involvement was one of the factors determining the level of resuscitation quality in COVID-19 patients.

### Perceived differences in COVID-19 resuscitation compared to non-COVID patients

Based on statements of 11 participants and the obtained 48 codes, the fourth super-ordinate theme in this study was “perceived differences in COVID-19 resuscitation compared to non-COVID patients”. Participants who had previously served on a pre-epidemic resuscitation team had different resuscitation experiences based on this theme. In this regard, three sub-ordinate themes were obtained:

#### PPE is a limit to effective resuscitation in COVID-19

“PPE is a limit to effective resuscitation in COVID-19”, mentioned by 5 participants, was the first sub-ordinate theme of perceived differences in COVID-19 resuscitation compared to non-COVID patients. Participants were more tired than those attending non-COVID patient resuscitations due to the full use of PPE. Also, due to using double covering, this feeling was more intense in female participants. They got tired sooner and felt exhaustion, oxygen deficiency, and intolerance to activity. As a result, they were not willing to use the complete PPE during resuscitation.

One of the 39-year-old supervisors, working in COVID’s emergency department since the onset of the epidemic, said:

*‘It’s strangling, and it’s lowering the quality of work, particularly for us. We feel exhausted during resuscitation. It’s not at all comparable to the resuscitation in non-COVID patients and comfort of the clothes there’. (Participant No. 18).*

Another 41-year-old nurse said:

*‘Using this equipment in resuscitation is tough. You may not believe that 5 minutes of resuscitation with this cover equals one hour of normal resuscitation’. (Participant No. 23).*

The use of PPE during resuscitation slowed the execution of orders, impaired mental planning for resuscitation, and disrupted the movement and communication between resuscitation team members. In this respect, a 40-year-old nurse with 16 years of experience working in various departments said:

*‘One becomes hypoxic with this equipment, one’s mind goes blank to think and plan resuscitation, even to execute orders. I feel that the resuscitation process and communication with a colleague is slowing, which also affect the resuscitation results’. (Participant No. 21).*

#### Delay in the resuscitation

The second sub-ordinate theme of perceived differences in COVID-19 resuscitation compared to non-COVID patients was “Delay in the resuscitation”, four participants mentioned. When patients in need of critical care arrived in the emergency department, some participants delayed life-saving actions until they had access to PPE or the presence of a nurse anesthetist for intubation. In contrast to non-COVID patients’ resuscitation experiences, even participants who were proficient enough in intubation did not volunteer to do so. This process delayed the onset of advanced life support for the patient. A 39-year-old supervisor with 16 years of nursing experience expressed:

*‘In the intubation discussion, no one touches the patient or intubates until the nurse anesthetist arrives with all of the necessary PPE. In previous emergencies, in non-COVID patients, I would intubate the patient many times before the doctor or anesthesia nurse arrived; however, I did not intubate a single case here, and this process delays the start of advanced life support’. (Participant No. 18).*

A 42-year-old nurse who worked in the COVID-19 emergency department said*:*

*‘For a patient who arrested out of the hospital and her family members bring her or him to the hospital, if we want to wear shields, gloves or scrubs or protective equipment, sometimes starting resuscitation is delayed, affecting the resuscitation process. However, this difference is less felt for patients who are already in the emergency room. The reason is that when we know the patient is ill and may be coded, we prepare for it’. (Participant No. 14).*

Because of their experience with delayed resuscitation, some resuscitation team members believe this method is involved in poor resuscitation outcomes in COVID-19 patients.

#### The futility of resuscitation in COVID-19

The third sub-ordinate theme of perceived differences in COVID-19 resuscitation compared to non-COVID patients was “the futility of resuscitation in COVID-19”, which was mentioned by 7 participants. For some participants, COVID labeling along with cardiac arrest was regarded as the patient’s death. Despite participating in the resuscitation of patients, some participants did not expect the patient to recovery and considered resuscitation a futile effort for the patient with COVID-19. In this regard, a 43-year-old emergency medicine specialist with 14 years of experience expressed regret for this attitude as the resuscitation team leader:

*‘We had a COVID-19 patient, but we did not perform proper resuscitation. The supervisor came and told us to resuscitate for two minutes. It means only compressing the chest for two minutes, and even if you didn’t intubate, there is no problem because the patient has COVID-19’. (Participant No. 16).*

Also, one of the 42-year-old nurses, who had a 16-year of experience in nursing, stated:

*‘Well, most of us have the same view in resuscitating this patient. Well, he has COVID-19; let’s finish it somehow. The patient with COVID who reached the stage of cardiac arrest,* i.e.*,‘expire”. (Participant No. 14).*

As a result, some participants regarded resuscitation of these patients as “slow code” and, in many cases, performed resuscitation solely to resolve the task or to avoid legal consequences.

## Discussion

This study aimed to explore the HCPs’ experiences of cardiopulmonary resuscitation in patients with COVID-19. After analyzing the data, four super-ordinate themes and nine sub-ordinate themes were extracted. The first super-ordinate theme of this study was the “Human aspect of care”. Based on this theme, the participants show a more compassionate effort to rescue patients and their sense of humanity led to prefer to help the critically ill patient rather than protect themselves. Although the outbreak of COVID-19, has created barriers and problems for HCPs to do CPR process perfectly, however, it has also created opportunities to flourish in the humanity dimension of care. Such experiences are a stimulus for the HCPs leading to more resuscitation efforts in patients with Covid-19. It seems the “altruistic acts” in other research and from nurses’ experiences in caring for COVID patients [18], emerged as the “human aspect of care” in our study. In line with these findings, another study showed that “Human care” was one of the sub-categories extracted from nurses’ experiences in providing care to patients with Covid-19. Because of this dimension, nurses risked their health to provide effective care to patients with Covid-19 [25].

The second super-ordinate theme of the study was “Perceived psychological effects of resuscitation in COVID-19”, which was developed regarding the Fear of infection and transmission to family members. According to this theme, resuscitation team members are in close contact with a patient and face a high risk of infection during resuscitation. Thus, they are afraid of becoming infected with the virus and spreading it to their family members. In this study, participants’ concerns about family members were consistent with the study of Lee et al. [26], especially among married participants and those who had children. Results of a study led to extracting the theme “negative emotions”, consisting of fear and anxiety, and concern for family members [18]. In addition, the results of the study of Ardabili et al. [16] showed high levels of stress, fear, and anxiety among HCPs in the early phases of the COVID-19 pandemic. It seems that despite the vaccination of team members, the virus’s strange behavior, particularly in the emergence of mutated waves and strains, long and close contact of resuscitation team members with a patient with COVID-19, has exposed them to such psychological effects. Therefore, early psychological intervention is particularly important to resuscitation team members in an epidemic. At the same time, it is important to establish early support systems [18], such as adequate supplies of protective materials, care services for resuscitation team members’ families, and establish facilities and take necessary measures to ensure that contaminants are not transmitted when the resuscitation team members return home.

“HCPs’ perception of factors affecting the resuscitation process in COVID-19” was the third super-ordinate theme of study. According to this theme, the underlying conditions and severity of the COVID patient’s pulmonary involvement influence the resuscitation process and quality. Evidence shows that the patient’s clinical condition is an motivating factor of HCPs in providing high quality resuscitation [27]. Based on the findings of a qualitative study “critical clinical conditions of the patient” was one of the sub-categories extracted from the experiences of participants associated with CPR barriers in non-COVID patients; meaning old age or underlying disease was reported as the risk factors resulting in ineffective CPR [28]. Andersen et al. (2015) in their study concluded that, old age and the high prevalence of underlying diseases may affect the behavior of resuscitation team members [29]. There are significant clinical and ethical challenges for HCPs in the absence of data that make it possible to adopt a clear policy to determine the usefulness of resuscitation in COVID-19 patients [8]. Such challenges lower the motivation of resuscitation team members and resuscitation quality in patients with underlying diseases or pulmonary involvement.

The fourth super-ordinate theme was perceived differences in COVID-19 resuscitation compared to non-COVID patients. According to this theme, participants have different experiences in COVID-19 resuscitation compared to non-COVID patients. Due to the first sub-ordinate theme in this theme, “PPE is a limit to effective resuscitation in COVID-19”. In this regard, partisipants experience early fatigue, activity intolerance, sweating, hypoxia, slowed communication with teammates, and impaired mental planning to resuscitate when using the complete PPE in resuscitation. Similar to ours, in a qualitative study conducted by Hoernke et al. [30], participants described PPE use during care as cumbersome and tedious. The slowness of care and stress in specific procedures such as intubation due to cumbersome protective equipment were alsoo participants’ experiences. According to other studies, nurses become tired and exhausted by wearing PPE for long time [17, 18, 25]. Negative pressure breathing masks (N95), on the other hand, increase the work of breathing, increase the amount of carbon dioxide in the dead space, and reduce the amount of oxygen in the dead space, all impairing the cognitive tasks [31, 32]. Hence, the effects mentioned above can disrupt HCPs’ performance and affect the quality and quantity of resuscitation. Given the need to use this equipment by resuscitation team members, it seems that the problem can be resolved to some extent by improving the ventilation of the resuscitation room.

The second sub-ordinate theme of perceived differences in COVID-19 resuscitation compared to non-COVID patients was “Delay in the resuscitation”. Based on this theme, some participants had experienced the delayed onset of resuscitation in COVID-19 patients. According to the American Heart Association’s interim guideline, delaying the start of resuscitation for a patient with COVID-19 seems reasonable to use PPE [8]. In line with these findings, the results of a study by Janatolmakan et al. [28], showed that “Delayed attendance of the CPR” was one of the sub-categories extracted from nurses’ experiences related to CPR barriers. In contrast to our findings, a quantitative study found no significant delay difference in alert time or the onset of the resuscitation between before and after the epidemic in the resuscitation process [7]. The results may originate from the weakness of retrospective studies due to documentation errors resulting from the recording of delayed resuscitation events, leading to a misunderstanding of COVID-19’s inherent lethality [33]. In addition, different results may be obtained when evaluating the delay trend in different departments of medical centers. Unlike the emergency department as our study context, in units like intensive care, where severely ill patients have advanced airways and staff is equipped with PPE, there is no reason to delay the onset of resuscitation.

“The futility of resuscitation in COVID-19” was the third sub-ordinate theme of the perceived differences in COVID-19 resuscitation compared to non-COVID-19 patients. This attitude affects the motivation to provide quality resuscitation to these patients. Feelings of resuscitation futility by the participants of our study have emerged as themes of “estimation of chances of survival” and “sense of providing futile care” in studies conducted by Assarroudi et al. (2017) and Ardebili et al. (2021), respectively [16, 27]. When dealing with cardiac arrest events, resuscitation team members mentally estimate the chances of survival based on previous experiences of similar resuscitation cases. Such an attitude can generate a sense of futility or the usefulness of resuscitation measures. In this way, the quality of resuscitation is affected by the attitude of the resuscitation team members, therefore, it is necessary to develop an atmosphere to nurture a positive attitude among HCPs for optimizing the outcomes of resuscitation COVID-19 patients.

## Limitations

The main limitation of the current study was due to the prevailing conditions caused by the crisis of the waves of COVID-19 at the time of the study. On the one hand, the work load of doctors and nurses and the reduction of their free time made it difficult for them to attend the interview sessions. On the other hand, the need to comply with health protocols during the interviews was a factor for limiting access to the facial language of the participants in the masked areas.

Also, at the beginning of the interview, some participants did not want to share some of their experiences of the facts related to the process of CPR in COVID-19 patients. This limitation was partially lifted by ensuring that information was kept confidential. Also, the principle of limitation in the generalizability of the results of qualitative studies is one of the limitations of our research.

## Conclusion

During resuscitation of COVID-19 patients, participants experienced a wide range of feelings and emotions. Exploring these experiences led to four super-ordinate and nine sub-ordinate themes. “Human aspects of Care”, “Perceived psychological effects of resuscitation in COVID-19”, “HCPs’ perceptions of factors affecting the resuscitation process in COVID-19”, and “Perceived differences in COVID-19 resuscitation compared to non-COVID patients” were super-ordinate themes. In the resuscitation process of patients with COVID-19, while some participants made more compassionate efforts to resuscitate these patients by communicating emotionally with them, others saw resuscitation as a futile attempt and did not believe in resuscitation in these patients. On the other hand, 1 year after the COVID epidemic, although initial fear decreased over time, with the onset of new picks, new fears and anxiety reappeared among HCPs. Therefore, comprehensive support should be provided to protect the mental health of resuscitation team members and improve CPR staff’s performance in the COVID-19 epidemic.

## Data Availability

The datasets used and/or analyzed during the current study are available from the corresponding author on reasonable request.

## Abbreviations

CPR: Cardiopulmonary resuscitation
IPA: Interpretative phenomenological analysis
WHO: World Health Organization
ROSC: Return of spontaneous circulation
IHCA: in-hospital cardiac arrest
BSc: Bachelor
MSc: Masters
GP: General Practitioner
PPE: Personal protective equipment
HCP: Healthcare provider
